# Supplementary material for: A Two-Sample Mendelian Randomization Analysis Investigates Associations Between Gut Microbiota and Celiac Disease
Source: Nutrients. 2020 May 14;12(5):1420. doi: 10.3390/nu12051420 (PMC7284592; doi:10.3390/nu12051420)
Supplement: Supplementary file 1 [file nutrients-12-01420-s001.pdf]

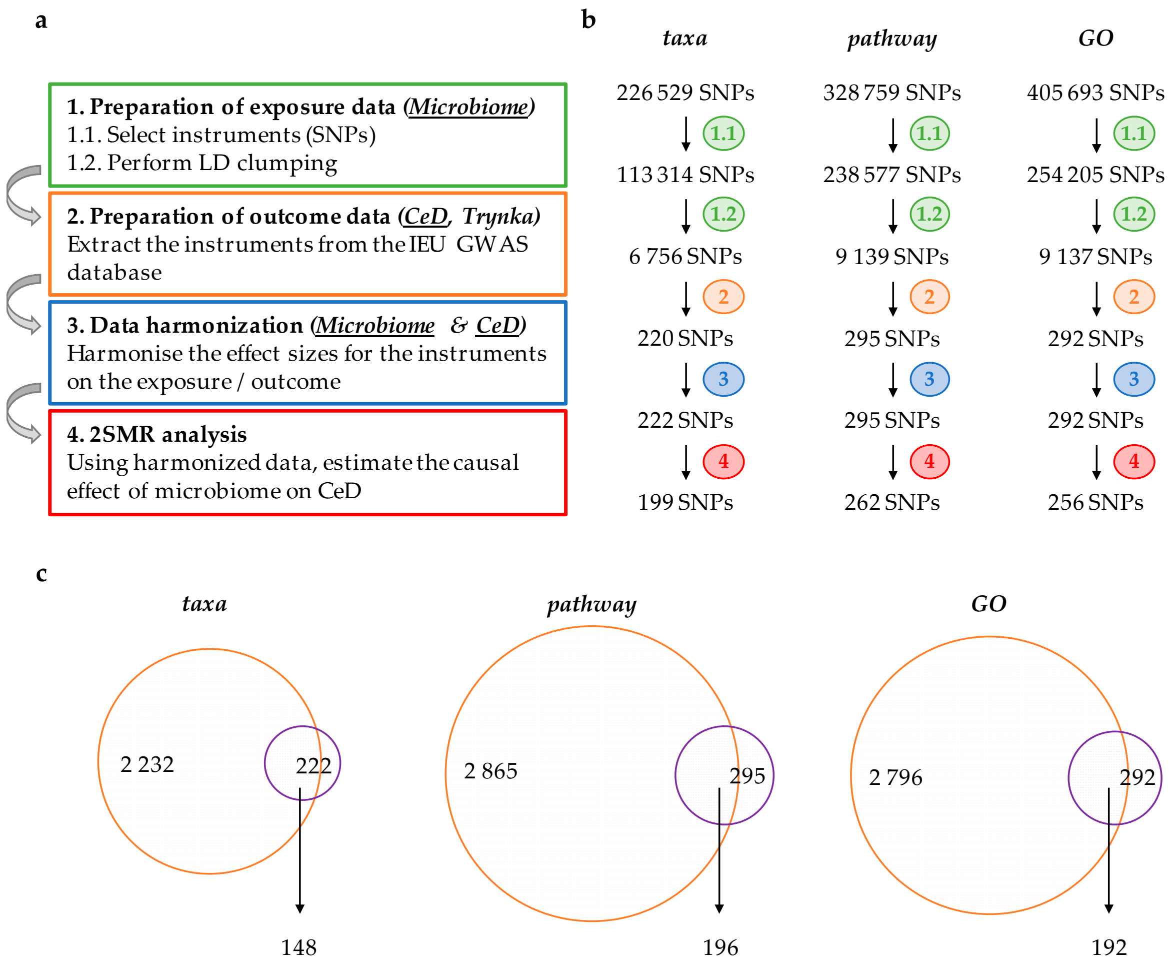

Figure S1. Schematic representation of 2SMR analysis using Bonder microbiome and Trynka CeD GWASs as exposure and outcome datasets, respectively. (**a**) Flowchart of step-by-step analysis: after preparing exposure data (Step 1), outcome data is extracted (Step 2), both datasets are harmonized (Step 3), and 2SMR analysis is performed (Step 4). (**b**) Diagram, representing the number of SNPs selected in each category (taxa, pathway, GO) after performing each step of the analysis. (**c**) Venn-diagrams, illustrating the number of SNPs in harmonized datasets derived from Bonder vs. Dubois (orange) or Bonder vs. Trynka (purple) analyses.
